# The Effect of Social Determinants and Socioeconomic Status on Laparoscopic Roux-En-Y Gastric Bypass for Weight Loss: An Analysis of the National Inpatient Sample

**DOI:** 10.1055/s-0041-1734030

**Published:** 2021-07-19

**Authors:** Supreet Singh, Jarot J. Guerra, Paige Lazar, Aziz M. Merchant

**Affiliations:** 1 Department of Surgery, Rutgers New Jersey Medical School, Newark, New Jersey

**Keywords:** bariatric surgery, surgical outcomes, health services research, obesity treatment, health disparities, laparoscopic Roux-en-Y gastric bypass

## Abstract

**Objective** In the United States, Black and Hispanic patients have a higher prevalence of obesity than Whites (49.6 vs. 44.8 vs. 42.2%, respectively). Despite higher rates of obesity among minority populations, bariatric surgery is performed at higher obesity levels in minorities than in Whites. This study examines the effects of various socioeconomic factors such as race, payer type, and income on the likelihood of undergoing laparoscopic Roux-en-Y gastric bypass (LRYGB) at class II versus class III obesity and their associated complications.

**Materials and Methods** National Inpatient Sample (NIS) from 2016 to 2017 was queried to identify patients at least 18 years of age with a concomitant diagnosis of class II or class III obesity who underwent LRYGB. We analyzed obesity level at the time of LRYGB (class II vs. class III), postoperative intestinal obstruction during the admission, and occurrence of any noninfectious complication related to the surgery as our main outcomes. A multivariate logistic regression model was utilized to assess the association between our outcomes and socioeconomic factors associated with the admission.

**Results** A total of 76,405 LRYGB operations were included. Out of this total, 83% (63,640) LRYGB operations were in class III obesity. Black patients had a lower rate (11.6%) of LRYGB procedures at class II obesity than White (17.6%) and Hispanic (18%) patients ( *p* < 0.001). Medicare, Medicaid, and lower income quartiles also showed lower rates of operation at class II obesity ( *p* < 0.001). Black patients were 29% (95% confidence interval [CI]: 0.61–0.83, *p* < 0.001) less likely than Whites to have a LRYGB procedure at class II obesity, they were 119% (95% CI: 1.17–4.11, *p* = 0.0014) more likely to suffer a postoperative intestinal obstruction, and they were 93% (95% CI: 1.31–2.84, *p* < 0.001) more likely to suffer a noninfectious complication.

**Conclusion** Socioeconomic disparities in the surgical management of severe obesity persist in the United States, especially for LRYGB. This study highlights multiple demographic factors that led to LRYGB at later obesity levels. Black patients were also more likely to be associated with postoperative complications during the admission. The determinants of health disparities in obese patients need to be examined further to reduce potential long-term morbidity and mortality in minorities. Further research is also required to identify the adverse effects of health disparities in patients with severe obesity and obesity-related comorbidities.

Health disparities in the surgical management of severely obese patients prevail in the United States. According to the most recent data from the 2017 to 2018 National Health and Nutrition Examination Surveys, Non-Hispanic Black and Hispanic patients have a higher age-adjusted prevalence of obesity compared with Non-Hispanic Whites (49.6 vs. 44.8 vs. 42.2%, respectively). Black patients also have a higher prevalence of severe obesity than Whites (13.8 vs. 9.3%). 1 Despite a greater prevalence of obesity, Non-Hispanic Black and Hispanic patients have been shown to utilize bariatric surgery less, constituting only 10% of bariatric surgery patients. 2 High quality evidence suggests that bariatric surgery is associated with better weight loss, 3 4 5 comorbid remission, 6 and cost-effectiveness per quality-adjusted life-year 7 compared with nonsurgical management of severe obesity. Bariatric surgery is additionally associated with low morbidity and mortality at accredited centers. 8 9 10 11 12 Likely the result of multiple factors, 13 14 15 minorities are not afforded equal access to these life-saving procedures. 16

Bariatric surgery is a key component of the contemporary management of severe obesity in the world. In the United States, the National Institutes of Health (NIH) published consensus guidelines 4 with eligibility criteria for bariatric surgery. The guidelines included various criteria including body mass index (BMI) of greater than or equal to 40 kg/m ^2^ or a BMI of 35 to 39.9 kg/m ^2^ with an obesity-related comorbidity such as type 2 diabetes mellitus, hypertension, and obstructive sleep apnea. High quality evidence suggests that bariatric surgery is associated with better weight loss, 5 6 7 comorbid remission, 8 and cost-effectiveness per quality-adjusted life-year 9 compared with nonsurgical management. In addition, when bariatric surgery is performed at accredited centers, it is associated with low morbidity and mortality. 10 11 12 13 14

Studies have shown that the outcomes of bariatric surgery are associated with certain socioeconomic disparities. Rios-Diaz et al have shown that Medicaid or Medicare insurance status, relative to commercial private insurance, was independently associated with readmission after bariatric surgery when they analyzed sleeve gastrectomy, gastric bypass, and gastric band together.

This disproportionate representation of minority patients in the population of bariatric surgical patients has been analyzed extensively as bariatric surgery has become a key component of the contemporary management of severe obesity. 13 17 The disparity in access has been identified, but few analyses have quantified it. This study aims to describe the disparity by comparing the BMI threshold of receiving laparoscopic Roux-en-Y gastric bypass (LRYGB) among White and non-White bariatric patients in the United States.

Not only underprivileged patients have less access to LRYGB, but also substantial evidence supports that their outcomes are poorer than other patients. Lessening the health disparity requires both increasing access to underprivileged populations and identifying and minimizing their morbidity. Studies have shown that Medicaid or Medicare insurance status, relative to commercial private insurance, was independently associated with readmission after bariatric surgery. 18 Others have found that African American patients were likely to have the longest length of hospital stays, highest complication rates, and least weight loss. 2 19 20 21 22 23 However, the majority of these analyses use 30-day readmission to assess complication rates. Research is lacking regarding acute complications of LRYGB in the minority population. Therefore, in addition to BMI analysis, this study examines whether socioeconomic factors could help predict immediate postoperative outcomes during the index admission.

## Materials and Methods

### Data Source

The National Inpatient Sample (NIS) from 2016 to 2017 was queried to conduct our retrospective analysis. The NIS is the largest publicly available all-payer inpatient database in the United States, containing more than 7 million hospitalizations per year and representing approximately 20% of all national inpatient discharges. The database is a part of the Health Care Cost and Utilization Project (HCUP), a family of databases sponsored by the Agency for Healthcare Research and Quality (AHRQ). All guidelines established by AHRQ were followed to account for the complex survey design of NIS data and ensure that proper weighted estimates and standard errors were calculated. Since the data in the analysis are de-identified and publicly available, this study was exempt from our institutional review board.

### Study Population

Patients at least 18 years of age with a concomitant diagnosis of class II (BMI = 35–40 kg/m ^2^ ) or class III (BMI > 40 kg/m ^2^ ) obesity who underwent LRYGB procedures from 2016 to 2017 were selected. International Classification of Diseases, Tenth Revision, Clinical Modification (ICD-10-CM) procedure codes were used to identify all admissions undergoing surgeries by using the primary procedure code, and ICD-10-CM diagnosis codes were used to classify admissions based on obesity class. The ICD-10-CM procedure and diagnosis codes used in the study are presented in Supplementary Table S1 (available in the online version). Due to the elective nature of these procedures, admissions with missing insurance information and admissions that were coded as self-pay, no charge, or other were excluded from the analysis. Admissions classified as Asian, Native American, or other were also excluded from the analysis due to their small sample size for LRYGB procedures. Lastly, any admissions with missing demographic data for age, sex, race, income quartiles, and payer type were also excluded from the study.

### Patient and Hospital Characteristics

The following admission-related demographics and hospital characteristics were identified: age, gender, race, median household income quartile, payer type, hospital teaching status, hospital region, hospital bed size, presence of concomitant diabetes and hypertension, and Elixhauser comorbidity index scores. Race was classified into three categories: Black, Hispanic, and White. Elixhauser comorbidity index scores were created by using the Elixhauser Comorbidity Software (Version 3.7) available on the HCUP website. The comorbidity index software generates two weighted scores (mortality score and readmission score) from a combination of 29 different comorbidities to capture the severity of overall comorbidity for each discharge.

### Outcomes

Outcomes of interest were the following: obesity level at the time of gastric bypass (class II vs. class III), postoperative obstruction during the admission, and occurrence of any noninfectious complication related to the surgery during admission. The ICD-10-CM codes used to define the outcomes are also reported in Supplementary Table S1 (available in the online version).

### Statistical Analysis

Univariate analysis was conducted by using a Rao-Scott Chi-square test for categorical and dichotomous variables, and a *t* -test for continuous variables. The adjusted multivariate logistic regression for our outcomes controlled for all patient demographics (age, gender, race, payer type, and income quartile), hospital level variables (region, location/teaching status, bed size), and severity of Elixhauser comorbidities (including diabetes and hypertension). An α level of 0.05 was considered statistically significant, and appropriate discharge weights were used to produce results representative of national estimates. As per HCUP guidelines, categorical variables are presented as frequencies with their relative percentages and continuous variables are listed as their mean ± standard error (SE). Logistic regression results were reported by using odds ratios (OR) and 95% confidence intervals (CI). All statistical analysis was performed by using SAS 9.4 software (SAS Institute Inc., Cary, NC) using their survey procedures.

## Results

### Univariate Analysis

The weighted sample was representative of 76,405 LRYGB procedures. Of all the procedures, 12,765 (16.7%) were performed at class II obesity and 63,640 (83.3%) were performed at class III obesity. Patient demographics showed significant variation in the timing of bariatric surgery with regards to obesity level. On average, patients who underwent LRYGB at class II obesity were older than patients who underwent the operation at class III obesity (49.1 vs. 44.2 years, *p* < 0.001). In terms of race, Black patients had a lower rate (11.6%) of LRYGB procedures at class II obesity than White (17.6%) and Hispanic (18%) patients ( *p* < 0.001). Admissions classified as Medicare and Medicaid also showed lower rates of operation at class II obesity when compared with admissions with commercial insurance ( *p* < 0.001, Table 1 ). The rate of LRYGB procedures at earlier obesity levels also increased as the median income quartile associated with the admission increased ( *p* < 0.001, Table 1 ). Discharges with hypertension and diabetes had higher rates of surgery at lower obesity than patients without those comorbidities ( Table 1 ). The only demographic variable that showed significant findings for complications was race. Black patients at discharge had a higher occurrence of postoperative intestinal obstruction (0.7%, *p* = 0.018) and a higher occurrence of noninfectious complications (1.6%, *p* < 0.001) relative to White and Hispanic discharges ( Table 1 ).

**Table 1:** Socioeconomic factors by class II and II obesity surgery, postoperative obstruction, and noninfectious complications of bariatric surgery

	Obesity level at time of bypass			Postoperative obstruction			Noninfectious complications of bariatric procedures		
	Class II BMI (35–40 kg/m ^2^ )	Class III BMI (> 40 kg/m ^2^ )	*p* -Value	Yes	No	*p* -Value	Yes	No	*p* -Value
Age	49.1 (0.2)	44.2 (0.1)	<0.001	45.6 (1.5)	45.0 (0.1)	0.7063	47.6 (1.0)	45.0 (0.1)	0.0103
Gender									
Male	2,230 (14.9%)	12,725 (85.1%)	0.0028	70 (0.5%)	14,885 (99.5%)	0.3830	135 (0.9%)	14,820 (99.1%)	0.9704
Female	10,535 (17.1%)	50,915 (82.9%)		220 (0.4%)	61,230 (99.6%)		550 (0.9%)	60,900 (99.1%)	
Race									
White	9,430 (17.6%)	44,095 (82.4%)	<0.001	165 (0.3%)	53,360 (99.7%)	0.0181	425 (0.8%)	53,100 (99.2%)	0.0004
Black	1,415 (11.6%)	10,820 (88.4%)		85 (0.7%)	12,150 (99.3%)		190 (1.6%)	12,045 (98.4%)	
Hispanic	1,920 (18%)	8,725 (82%)		40 (0.4%)	10,605 (99.6%)		70 (0.7%)	10,575 (99.3%)	
Expected primary payer									
Medicare	2,110 (17.2%)	10,175 (82.8%)	<0.001	40 (0.3%)	12,245 (99.7%)	0.5428	150 (1.2%)	12,135 (98.8%)	0.1458
Medicaid	1,875 (12.2%)	13,540 (87.8%)		75 (0.5%)	15,340 (99.5%)		120 (0.8%)	15,295 (99.2%)	
Private insurance	8,780 (18%)	39,925 (82%)		175 (0.4%)	48,530 (99.6%)		415 (0.9%)	48,290 (99.1%)	
Patient income									
Quartile 1	2,950 (14.4%)	17,520 (85.6%)	<0.001	100 (0.5%)	20,370 (99.5%)	0.0817	215 (1.1%)	20,255 (98.9%)	0.7104
Quartile 2	3,365 (15.9%)	17,770 (84.1%)		80 (0.4%)	21,055 (99.6%)		185 (0.9%)	20,950 (99.1%)	
Quartile 3	3,555 (16.7%)	17,715 (83.3%)		40 (0.2%)	21,230 (99.8%)		175 (0.8%)	21,095 (99.2%)	
Quartile 4	2,895 (21.4%)	10,635 (78.6%)		70 (0.5%)	13,460 (99.5%)		110 (0.8%)	13,420 (99.2%)	
Hypertension									
No	4,950 (15.4%)	27,270 (84.6%)	0.0007	120 (0.4%)	32,100 (99.6%)	0.9051	255 (0.8%)	31,965 (99.2%)	0.2527
Yes	7,815 (17.7%)	36,370 (82.3%)		170 (0.4%)	44,015 (99.6%)		430 (1%)	43,755 (99%)	
Diabetes without chronic complications									
No	8,755 (15.6%)	47,280 (84.4%)	<0.001	225 (0.4%)	55,810 (99.6%)	0.4562	520 (0.9%)	55,515 (99.1%)	0.5032
Yes	4,010 (19.7%)	16,360 (80.3%)		65 (0.3%)	20,305 (99.7%)		165 (0.8%)	20,205 (99.2%)	
Diabetes with chronic complications									
No	11,230 (16.2%)	58,170 (83.8%)	<0.001	275 (0.4%)	69,125 (99.6%)	0.2909	595 (0.9%)	68,805 (99.1%)	0.1300
Yes	1,535 (21.9%)	5,470 (78.1%)		15 (0.2%)	6,990 (99.8%)		90 (1.3%)	6,915 (98.7%)	
Elixhauser Index Scores									
Mortality score	−0.1 (0.1)	0.0 (0.1)	0.0925	2.0 (0.8)	0.0 (0.1)	0.0135	1.8 (0.5)	0.0 (0.1)	<0.001
Readmission score	8.9 (0.2)	8.5 (0.2)	0.0326	10.1 (1.2)	8.5 (0.2)	0.1894	10.5 (1.0)	8.5 (0.2)	0.0372

Abbreviation: BMI, body mass index.

### Multivariate Logistic Regression Analysis

After simultaneously controlling for patient demographics, hospital level characteristics, and the comorbidity severity of discharges, the logistic regression yielded similar findings to our univariate analysis ( Table 2 ). Black patients were 29% (95% CI: 0.61–0.83, *p* < 0.001) less likely and Hispanics were 24% (95% CI: 1.08–1.43, *p* = 0.003) more likely than Whites to have a LRYGB procedure at class II obesity instead of class III obesity. Similarly, Medicare was associated with a 41% (95% CI: 0.52–0.68, *p* < 0.001) lower likelihood and Medicaid was associated with a 29% (95% CI: 0.63–0.82, *p* < 0.001) lower likelihood of LRYGB at class II obesity. Admissions in the first three income quartiles were all less likely to have LRYGB at class II obesity than the admissions in the highest income quartile ( *p* < 0.001, Table 2 ). Black patients were also 119% (95% CI: 1.17–4.11, *p* = 0.0014) more likely to suffer a postoperative obstruction and 93% (95% CI: 1.31–2.84, *p* < 0.001) more likely to suffer another noninfectious complication in comparison to their White counterparts. Lastly, admissions in the third income quartile were also associated with a lower likelihood of postoperative obstruction relative to admissions in the highest income quartile ( Table 2 ).

**Table 2:** Association of socioeconomic factors with class II obesity surgery, postoperative obstruction, and noninfectious complications of bariatric surgery

	Surgery performed at class II obesity BMI (35–40 kg/m ^2^ )		Postoperative obstruction		Noninfectious complications of bariatric procedures	
	OR (95% CI)	*p* -Value	OR (95% CI)	*p* -Value	OR (95% CI)	*p* -Value
Age	1.04 (1.04–1.05)	<0.001	1.01 (0.99–1.03)	0.4368	1.02 (1.00–1.03)	0.0495
Gender						
Male	Reference		Reference		Reference	
Female	1.36 (1.21–1.52)	<0.001	0.76 (0.41–1.44)	0.4025	1.08 (0.67–1.74)	0.7398
Race						
White	Reference		Reference		Reference	
Black	0.71 (0.61–0.83)	<0.001	2.19 (1.17–4.11)	0.0142	1.93 (1.31–2.84)	<0.001
Hispanic	1.24 (1.08–1.43)	0.0025	1.23 (0.55–2.77)	0.6171	0.88 (0.52–1.50)	0.6496
Expected primary payer						
Medicare	0.59 (0.52–0.68)	<0.001	0.74 (0.32–1.71)	0.4754	1.08 (0.68–1.73)	0.7373
Medicaid	0.71 (0.63–0.82)	<0.001	1.32 (0.66–2.63)	0.4357	0.89 (0.56–1.42)	0.6316
Private insurance	Reference		Reference		Reference	
Patient income						
Quartile 1	0.74 (0.64–0.85)	<0.001	0.79 (0.37–1.67)	0.5379	1.25 (0.69–2.26)	0.4585
Quartile 2	0.79 (0.69–0.91)	<0.001	0.70 (0.33–1.47)	0.3436	1.12 (0.63–2.00)	0.6918
Quartile 3	0.79 (0.69–0.91)	0.0010	0.34 (0.14–0.83)	0.0182	1.00 (0.57–1.78)	0.9899
Quartile 4	Reference		Reference		Reference	

Abbreviation: BMI, body mass index; OR, odds ratio.

Note: Controls for hospital level variables and severity of comorbidities in addition to the variables shown above.

## Discussion

The study suggests that African Americans are most affected by access barriers to LRYGB surgery. As seen in the univariate and multivariate analyses, Black patient admissions were disproportionately less likely to get early LRYGB at class II obesity in comparison to their White and Hispanic counterparts. Additional differences were observed for insurance status and patient income quartiles. Discharges associated with Medicare, Medicaid, and lower income quartiles were also less likely to undergo a LRYGB at class II obesity relative to undergoing the procedure at class III obesity.

Despite increases in coverage for bariatric surgery by state employee programs and Medicaid, 24 25 study results still show low utilization of bariatric surgery for patients who are on governmental insurance plans. Some studies suggest the major limitation in delivering basic 7 26 and comprehensive 27 28 obesity-related surgery is limited reimbursement. In addition, several insurance plans require a 6-month minimum of supervised medical weight management, incurring additional cost, delaying treatment, and contributing to patient attrition. 14 Hennings et al found that delays and insurance rejection among the publicly insured seeking bariatric procedures lead to a threefold increase in mortality. 17

The NIH eligibility criteria 29 only allows class II obesity to receive surgery if they have a related comorbidity. Therefore, disease burden bears influence on which patients receive LRYGB at this lower BMI. Black and Hispanic patients have higher disease burden than their white counterparts and so would be expected to represent a larger percentage of LRYGB performed at class II obesity. The same is expected of Medicare and Medicaid patients and those in the lowest income quartiles, yet they similarly represent a low portion of these early interventions. In addition, the multivariate regression takes into account comorbidity severity, minimizing this effect on the data.

Socioeconomic disparities in elective surgery for minorities have been well documented in literature, and this study adds similar findings about elective surgery to existing literature for LRYGB. However, this study is the first study to use a nationally representative database to assess timeline with regards to obesity level and the performance of LRYGB.

Postoperative complications in bariatric surgery were also associated with race in the study cohort. Black patients were noted to have a statistically significant association with greater likelihood for postoperative intestinal obstruction compared with Hispanics and Whites (0.7 vs. 0.4 vs. 0.3%), respectively. Black patients were also associated with an increased likelihood of noninfectious postoperative complications compared with Whites and Hispanics (1.6 vs. 0.8 vs. 0.7%), respectively. Wood et al reported significantly higher rate of overall complications at 30 days in Black patients than in white patients. 20 Several studies found higher rates in readmissions among black patients as well as the publicly insured and those in lower income quartiles 18 19 20 21 22 23 30 Some studies have also reported increase in overall complications and no difference in mortality, 21 increase in mortality but no difference in complications, 22 and others have reported no racial differences and postoperative adverse effects. 23 31

This study's focus on LRYGB, despite a trend away from LRYGB in favor of sleeve gastrectomy in the United States, 19 30 is a matter of both clinical and statistical consideration. Analyses comparing the two techniques posit that BMI is likely to influence procedure choice, as LRYGB has proven to result in greater long-term weight loss. 19 30 32 To eliminate this confounding factor, as BMI is this study's main outcome of interest, LRYGB was assessed alone. Additionally, a reason for sleeve gastrectomy's growing popularity is its low complication rate 19 ; it has a better safety profile in the first 30 days postoperatively than LRYGB. 32 Bariatric surgery in general already has low complication rates, 18 and so there were too few complications in the index admission compared with all sleeve gastrectomies performed to make a statistically sound argument.

The data used from the NIS in our study has inherent limitations. Given the nature of administrative data, one of the limitations is the inability to account for possible miscoding of data points. Second, the NIS is an admission only database and does not provide any follow-up data for the admission after discharge. Therefore, it is important to highlight that morbidity and mortality rate in any NIS analyses only represent postoperative complications and mortality that occur during the index admission. The inability to capture readmission data can potentially decrease the number of complications identified in our study. Though our regression controlled for hospital level factors and severity of patient comorbidities, there also is no way to control for the variability in the experience of the operating room surgeons using our dataset. Lastly, though we control for patient comorbidities to the greatest extent allowed by the data, information on the severity of comorbidities is lacking in NIS. Despite the limitations of our data, our analysis still provides valuable insight into socioeconomic factors of receiving bariatric surgery. Further research needs to be conducted to examine long-term effects on obese patients, especially ones who are Black since they suffer delays in accessing bariatric surgery.
