# Supplementary material for: The Effect of Social Determinants and Socioeconomic Status on Laparoscopic Roux-En-Y Gastric Bypass for Weight Loss: An Analysis of the National Inpatient Sample
Source: Surg J (N Y). 2021 Jul 19;7(3):e147–53. doi: 10.1055/s-0041-1734030 (PMC8289683; doi:10.1055/s-0041-1734030)
Supplement: Supplementary file 1 — Supplementary Material [file 10-1055-s-0041-1734030-s2000030oa.pdf]

Supplementary Table S1 International classification of diseases, tenth revision, clinical modification diagnosis and procedure codes

Diagnosis/procedure	Diagnosis code
Roux-en-Y gastric bypass	0D164XX
Class II obesity	Z68.35-Z68.40
Class III obesity	Z68.40 or more
Postoperative obstruction	K95.3X
Noninfectious complications of bariatric procedures	K95.89

 © 2021. The Author(s).
